# Novel Sodium Carbonate Activation for Manufacturing Sludge-Based Biochar and Assessment of Its Organic Adsorption Property in Treating Wool Scouring Wastewater

**DOI:** 10.3390/toxics13040256

**Published:** 2025-03-29

**Authors:** Wanru Zhang, Hongrong Huang, Zhen Cao, Shuyu Kang, Xueqing Shi, Weiwei Ma, Harsha Ratnaweera

**Affiliations:** National and Local & Joint Engineering Research Center for Urban Sewage Treatment and Resource Recycling, School of Environmental and Municipal Engineering, Qingdao University of Technology, 11 Fushun Road, Qingdao 266033, China; wanru086@gmail.com (W.Z.); 19882107978@163.com (H.H.); 13906437699@163.com (Z.C.); ksy0225@163.com (S.K.); shixq85@163.com (X.S.)

**Keywords:** acid modification, adsorption property, sludge-based biochar (SB), sodium carbonate activation, wool scouring wastewater

## Abstract

Under the concept of green and low-carbon development, efficient and environmentally friendly biochar preparation methods have attracted much attention. This study assessed a novel sodium carbonate activator combined with acid modification for sludge-based biochar (SB) production and its adsorption of organics in wool scouring wastewater. Under 600 °C, the optimal carbonization temperature, the residual weight percentage of biochar carbonized material increases from 27% to 73% after Na_2_CO_3_ activation compared to ZnCl_2_ activation. Compared to HCl-modified ZnCl_2_-activated biochar (Zn-Cl-SB), HCl-H_2_SO_4_-modified Na_2_CO_3_-activated biochar (Na-Cl/S-SB) had a specific surface area of 509.3 m^2^/g, and the average mesopore size was 7.896 nm, with micropore volume and specific surface area increasing by 83.3% and 79.8%, respectively. Meanwhile, the C-O oxygen-containing functional groups and pyrrole nitrogen-containing functional groups were significantly increased. Na-Cl/S-SB exhibited an excellent adsorption performance for organic matter in wool scouring wastewater, with a maximum adsorption capacity of 168.3 mg/g. Furthermore, the adsorption process followed the pseudo-second-order kinetic model. Three-dimensional fluorescence spectrum analysis showed that Na-Cl/S-SB had a strong adsorption capacity for aromatic protein analogs, proteins containing benzene rings, and dissolved microbial by-products in wool scouring wastewater. This study will serve as a guideline for the green synthesis of SB while improving its ability to adsorb pollutants.

## 1. Introduction

Wool scouring wastewater contains high levels of fats, oils, and complex organics, posing environmental risks if treated improperly [1,2]. Traditional biological and advanced treatment methods struggle with its unique composition. To address these challenges, as a novel adsorbent material, sludge-based biochar (SB) has gained significant attention for its preparation methods and potential applications [3]. Traditional SB preparation methods mainly include physical and chemical activation [4]. The physical activation method uses inert gases or gas mixtures (e.g., H_2_O, CO_2_, air) to erode the carbonized material’s surface, removing residual tar and C-H compounds. This process effectively creates pores [5]. In contrast, the chemical activation method typically involves the pretreatment of sludge with chemical reagents such as ZnCl_2,_ Na_2_CO_3_ and H_3_PO_4_ to increase the pore structure and surface area of biochar [6,7,8], followed by pyrolysis to remove organics, leaving a carbon-rich solid residue [9]. However, there are still some challenges in the preparation of SB. For instance, since June 2011, China’s National Development and Reform Commission (NDRC) has banned ZnCl_2_-based production due to pollution concerns, highlighting the need for sustainable alternatives [10,11,12]. 

Researchers have recently explored alternative activating agents, such as KOH, NaOH, and composite activations [13,14,15]. In recent years, Na_2_CO_3_ activation of materials has become a focal point of numerous studies. Na_2_CO_3_, as a green chemical reagent, has emerged as a promising activator. Compared to traditional chemical activators, Na_2_CO_3_ is more preferable as it is readily available, fully soluble in water, poses minimal environmental hazards, and does not waste zinc during the activation process [16]. Additionally, alkaline activators like Na_2_CO_3_ can neutralize the acidity of wastewater, reducing secondary pollution [17,18]. The chemical activation of biochar derived from rice husks [19] and oil palm leaves [16] using Na_2_CO_3_ can effectively enhance its porosity and specific surface area, thereby improving its adsorption capacity for organic dyes [20]. Additionally, Shao et al. reported that Na_2_CO_3_ activation enhances biochar’s adsorption capacity for pollutants [21]. This structural enhancement provides more active sites for pollutant binding, further boosting the efficiency of biochar in wastewater treatment. Moreover, Niu et al. demonstrated that Na_2_CO_3_-modified biochar exhibits excellent removal efficiency for antibiotics [22]. Furthermore, Na_2_CO_3_ activation can significantly increase the functional groups on biochar, thereby enhancing the adsorption capacity of biochar for organic pollutants [23].

SB has shown significant progress in treating organic pollutants in real wastewater [24,25]. It effectively removes dyes, antibiotics, neonicotinoid pesticides, and other contaminants from industrial wastewater [26,27,28]. For example, SB derived from municipal sludge and bamboo waste achieved 69.7% and 80.1% removal of phenol and total cyanide in biologically treated coking wastewater. It also demonstrated over 91% removal efficiency for perfluoroalkyl and polyfluoroalkyl substances (PFASs) [29], highlighting its potential as an efficient adsorbent in wastewater treatment [30].

However, optimizing and improving the performance of SB using green, environmentally friendly, and cost-effective preparation methods, and enhancing its adsorption capacity for various organic pollutants in real wastewater remains a critical challenge [31]. This study explores SB’s surface area, pore structure, functional groups, and elemental composition using Na_2_CO_3_ activation combined with HCl and H_2_SO_4_ modification. Furthermore, this study explores the adsorption performance of the novel SB for organic pollutants in real wool scouring wastewater, providing insights for optimizing SB modification and improving its efficiency in wastewater treatment.

## 2. Materials and Methods

### 2.1. Preparation of Sludge-Based Biochar

The sludge used in this experiment was collected from the secondary sedimentation tank of a local wastewater treatment plant (Qingdao, China). The concentrations of mixed liquor suspended solids (MLSSs) and mixed liquor volatile suspended solids (MLVSSs) were 7.83 g/L and 4.35 g/L, respectively. First, the sludge was centrifuged and dried to a constant weight in an oven at 105 °C. Then, a mortar and pestle were used to thoroughly grind the dried sludge until it passed through a 100-mesh sieve, producing uniformly sized dry sludge particles. A certain amount of dry sludge was weighed and mixed with 4 mol/L sodium carbonate activator at a solid-to-liquid ratio of 1:2. The mixture was stirred and mixed thoroughly and soaked for 24 h. Then, it was subjected to oven-drying at 105 °C until it reached a stable weight, followed by particle size refinement using a 100-mesh sieve to prepare the carbonization precursor. The carbonization precursor was subsequently heated in a controlled environment under a N_2_ atmosphere, with a heating rate of 10 °C per minute, carbonization temperature at 600 °C, and a final temperature holding time at 120 min. The carbonized material obtained from the tubular furnace (Taiste MFLC-36/10P) was modified by acid. The acid modifiers were 1 mol/L sulfuric acid and 1.64 mol/L hydrochloric acid (5% mass fraction). The carbonized material was mixed with the acid modifiers at a solid-to-liquid ratio 1:10 and shaken thoroughly in a constant-temperature oscillator at 25 °C for 12 h. The SB was washed with ultrapure water until the pH of the washing liquid was close to neutral, and then dried at 150 °C to obtain sodium carbonate-activated and acid-modified SB. Composite acid-modified SB was obtained by repeating the process with different modifiers for a second modification. The SB samples activated with Na_2_CO_3_ and modified with H_2_SO_4_ and HCl were labeled as H_2_SO_4_-modified Na_2_CO_3_-activated biochar (Na-S-SB) and HCl-modified Na_2_CO_3_-activated biochar (Na-Cl-SB), respectively. The SB sample activated with Na_2_CO_3_ and subjected to composite modification using HCl and H_2_SO_4_ was labeled as Na-Cl/S-SB. The Zn-Cl-SB sample as a control sample was prepared following a similar procedure. The dried sludge was thoroughly mixed with a 4 mol/L ZnCl_2_ solution at a solid-to-liquid ratio of 1:2, followed by continuous stirring and impregnation for 24 h. The subsequent drying, carbonization, and acid modification processes (HCl modified) were conducted according to the protocols described for the sodium-carbonate-activated samples.

### 2.2. Characterization Methods of Sludge-Based Biochar

The SB samples were characterized comprehensively using multiple analytical methods. Thermogravimetric analysis (TGA) and derivative thermogravimetry (DTG) were performed with a thermogravimetric analyzer (Jiezhun Instruments, TGA-1150, Shanghai, China) under a N_2_ atmosphere with a gas flow rate of 50 mL/min, a heating rate of 10 °C/min, and a final pyrolysis temperature of 800 °C. The porous structure parameters, including specific surface area, micropore volume, and pore size distribution, were determined by nitrogen adsorption–desorption isotherms at 77 K using gaseous nitrogen (N_2_) as the adsorbate. Before analysis, the samples were degassed under a vacuum at 300 °C for 4 h. The specific surface area was calculated using the Brunauer–Emmett–Teller (BET) method, micropore volume was derived from the t-plot method, and pore size distribution was analyzed using the Barrett–Joyner–Halenda method with a fully automatic surface area and porosity analyzer (Micromeritics, ASAP 2460, Norcross, GA, USA). X-ray diffraction (XRD) was employed with an X-ray Diffraction Analyzer (Malvern, Panalytical PANalytical X’Pert 3, Almelo, NL, USA) to investigate the crystalline structure of SB samples, using α radiation with a scanning angle range of 10–80° and a scanning speed of 0.033 s/step. Surface functional groups were identified by Fourier-transform infrared spectroscopy (Thermo Scientific, Nicolet IS50, Waltham, MA, USA) in the range of 4000–400 cm^−1^, and surface chemical composition was characterized using X-ray photoelectron spectroscopy (Thermo Fisher Scientific, EscaLab 250Xi, Waltham, MA, USA).

### 2.3. Adsorption Experiments of Sludge-Based Biochar

The water sample was collected from a wool scouring wastewater treatment plant, with a COD concentration of 2165 mg/L, an NH_3_-N concentration of 235 mg/L, and a pH of 5. The pH of the wastewater was adjusted to 9, which facilitates the precipitation of fats and lipids, preventing them from clogging the biochar’s pores. Additionally, the alkaline environment enhances the dispersion of organic compounds, promoting adsorption efficiency. After settling, 10 mL of the supernatant was taken and placed into a 50 mL beaker. A certain amount of sludge-based biochar (0.05 g) was added and then placed in a constant-temperature shaker at 200 r/min and 23 °C for 2 h to adsorption. The water sample was then filtered through a 0.45 µm membrane, and the COD concentration was measured using the potassium dichromate method [32].

The adsorption process of SB was analyzed using pseudo-first-order (PFO) and pseudo-second-order (PSO) adsorption kinetics models [33].

The linear equation of PFO adsorption kinetics is shown in Equation (1):

(1) $$\ln\left(1-\frac{q_{t}}{q_{e}}\right)=-k_{1}t$$

where:

$q_{t}$ is the amount of adsorbate adsorbed by the adsorbent at time t (mg·g⁻^1^);

$q_{e}$ is the amount of adsorbate adsorbed at adsorption equilibrium (mg·g⁻^1^);

$k_{1}$ is the adsorption rate constant (1/min).

The linear equation of PSO adsorption kinetics is shown in Equation (2):

(2) $$\frac{t}{q_{t}}=\frac{1}{k_{2}q_{e}^{2}}+\frac{t}{q_{e}}$$

where:

$q_{t}$ is the amount of adsorbate adsorbed by the adsorbent at time t (mg·g⁻^1^);

$q_{e}$ is the amount of adsorbate adsorbed at adsorption equilibrium (mg·g⁻^1^);

$k_{2}$ is the adsorption rate constant (1/min).

In Equations (1) and (2), $q_{e}$ is calculated by subtracting the COD value of the equilibrium water sample from the initial COD value, multiplying the difference by the volume of the solution (L), and then dividing by the mass of the biochar (g). Similarly, $q_{t}$ is determined by subtracting the COD value of the water sample at time t from the initial COD value, with the subsequent calculation steps being the same as for $q_{e}$. Accordingly, the unit is expressed as mg/g, which represents the mass of organic pollutants adsorbed per gram of biochar.

Three-dimensional fluorescence excitation–emission matrix (EEM) spectroscopy was used with fluorescence spectrometer (HORIBA, FluoroMax-4, Edison, NJ, USA) to analyze the changes in organic matter composition in wool scouring wastewater before and after adsorption. The excitation wavelength ranged from 200 to 400 nm with a 5 nm sampling interval, while the emission wavelength spanned 220 to 550 nm, also with a 5 nm sampling interval. The measurements were conducted with a slit bandwidth of 5 nm for both excitation and emission, and spectra were recorded at a scanning speed of 12,000 nm/min [34]. To evaluate the transformation of organic matter in wool scouring wastewater before and after adsorption, ultraviolet–visible (UV-Vis) spectroscopy (HACH, DR6000, Loveland, CO, USA) was used to analyze absorbance variations at specific wavelengths, providing insights into changes in molecular weight distribution. The measurements were conducted using a UV-Vis spectrophotometer within a scanning range of 800–200 nm. [35]. Additionally, to further characterize dissolved organic matter (DOM), we introduced the E_2_/E_3_ and E_2_/E_4_ indices for analysis. E_2_/E_3_ represents the UV-Vis absorbance ratio at 250 nm and 365 nm, while E_2_/E_4_ is defined as the absorbance ratio at 240 nm and 420 nm.

## 3. Results and Discussion

### 3.1. Optimization of Carbonization Temperature for Sludge-Based Biochar

**Figure 1:**
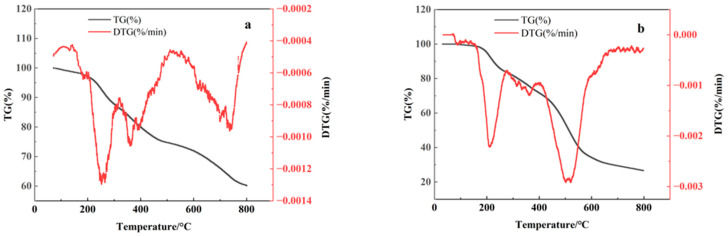
Thermogravimetric curves of carbonization precursor: (**a**) Na_2_CO_3_-activated carbonization precursor; (**b**) ZnCl_2_-activated carbonization precursor.

To determine the optimal carbonization temperature, thermogravimetric analysis (TGA) was performed on dried activated sludge samples pretreated with Na_2_CO_3_ activator. Figure 1a illustrates that the sludge undergoes four distinct stages of weight loss during the process. The first stage (30–250 °C) releases free and bound water in sludge, with minimal weight loss. The second and third stages (250–580 °C) involve significant weight loss due to the decomposition of organic matter and volatile components, which are degradable organic substances such as cellulose, hemicellulose, and lignin that are abundant in sludge generated during wastewater treatment. The second stage (250–350 °C) is mainly associated with the decomposition of hemicellulose. The third stage (350–580 °C) corresponds to the decomposition of cellulose and lignin [36]. The fourth stage (580–700 °C) corresponds to the pyrolysis of refractory residues. The rapid pyrolysis after 700 °C may indicate the increased proportion of sludge ash at high temperatures, which hinders pore formation and accelerates volatile release [37]. In Figure 1b, the DTG curve exhibits a faster decomposition rate compared to Figure 1a. Additionally, pyrolysis gradually stabilizes above 600 °C, indicating that the sample may have a higher ash content, which inhibits pore structure formation and reduces the release of volatile components. Considering this, 600 °C was selected as the optimal carbonization temperature. 

A comparison of Figure 1a,b shows that the residual weight percentage of biochar carbonized at 600 °C increased from 27% to 73% after Na_2_CO_3_ activation, an increase of 170%. This suggests that Na_2_CO_3_ activation is crucial for enhancing both the yield and structural stability of biochar.

### 3.2. Structural Characteristics Analysis

#### 3.2.1. Specific Surface Area and Pore Size Distribution

As shown in Table 1, although the specific surface areas of Na-S-SB and Na-Cl-SB are lower than that of Zn-Cl-SB, their average pore size and mesopore size are larger than those of Zn-Cl-SB. This indicates that pore size distribution is primarily concentrated in the micropore and more extensive mesopore ranges. Related studies suggest that this phenomenon is caused by the crosslinking and polycondensation of organic matter during the acid modification process. Zinc chloride in hydrochloric acid solution corrodes the dry sludge during activation and drying, initially forming a specific pore structure, further modified by acid treatment [38]. Moreover, residual sodium carbonate reacts with the acid modification solution, weakening the modification effect and leading to incomplete modification. As a result, Zn-Cl-SB exhibits a higher specific surface area than Na-S-SB and Na-Cl-SB.

In addition, as shown in Table 1, the specific surface area of Na-Cl/S-SB is 509.3 m^2^/g, which is higher than that of Zn-Cl-SB, Na-S-SB, and Na-Cl-SB. Compared to Zn-Cl-SB, the micropore volume increased by 83.3%, the micropore specific surface area increased by 79.8%, the average mesopore size increased by 51.3%, and the total pore volume increased by 28.0%. The percentage of micropores in the total pore volume of Na-Cl/S-SB is the highest, 5.9% higher than that of Zn-Cl-SB. This indicates that composite acid modification can significantly increase the specific surface area of SB. The results suggest that secondary acid modification dissolves residual components on the surface of biochar that were not decomposed at high temperatures or dissolved during the initial acid modification. These residual components may include activator remnants, inorganic salts, or ash trapped within SB pores. Composite acid modification enhances the removal of entrapped components within the biochar structure, some of which may contribute to gas evolution, leading to improved porosity and surface area. The findings show that the strong dissolving effect of composite acid modification broadens the pore size of SB, enhances the pore-opening effect, and generates more micropores. The increased specific surface area of SB improves its adsorption capacity of organic pollutants from wastewater, while the micropores are more effective at adsorbing small molecular compounds [34].

Table 2 presents the BET specific surface areas of biochar that different activators activated. Compared to other activators, Na_2_CO_3_ activation resulted in a significantly higher specific surface area, indicating its effectiveness in enhancing the porosity of sludge-based biochar. This improvement is critical for adsorption applications as surface area is key in pollutant removal efficiency. The findings highlight the potential of Na_2_CO_3_ as a promising activator for modifying sludge-based biochar, offering a scalable and environmentally friendly approach to optimizing biochar properties. 

#### 3.2.2. Elemental Composition

**Figure 2:**
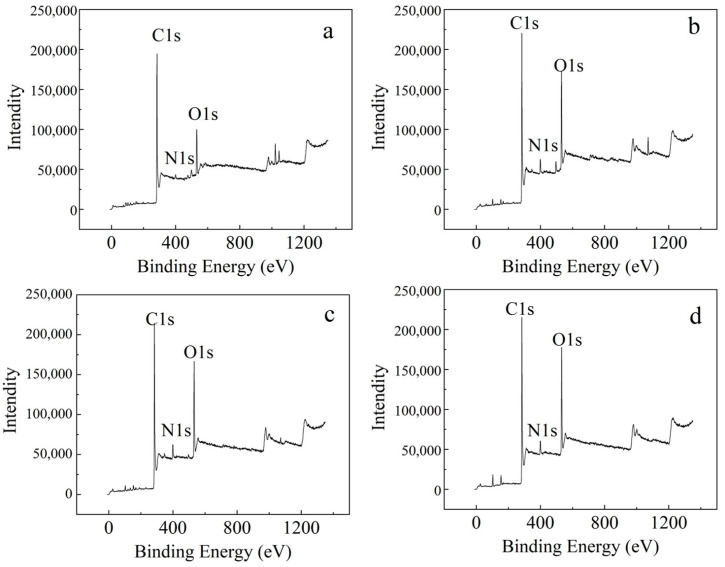
XPS full spectra of SB: (**a**) Zn-Cl-SB; (**b**) Na-Cl-SB; (**c**) Na-S-SB; (**d**) Na-Cl/S-SB.

To gain a deeper understanding into the surface elemental composition and functional group distribution of SB, an XPS analysis was conducted. As shown in Figure 2, the XPS spectra indicate that the main elements in SB prepared using different methods include C1s, N1s, and O1s. To investigate the impact of different preparation methods on the functional group structure and elemental composition of SB, this study utilized advantage curve-fitting and peak deconvolution techniques to mathematically process the C1s, N1s, and O1s peaks in the XPS spectra of different SB samples, analyzing the types and quantities of functional groups. As shown in Table 3, the proportion of nitrogen-containing functional groups in Na-Cl/S-SB, Na-Cl-SB, and Na-S-SB increased by 2.39–3.13% compared to Zn-Cl-SB. Related studies have shown that ZnCl_2_ activation causes most of the nitrogen to volatilize as N_2_, NH_3_, and HCN, with only a tiny portion of heterocyclic nitrogen transferring into the pyrolysis products. In contrast, the reaction between sodium carbonate solution and organic matter in the sludge promotes nitrogen retention, forming nitrogen-doped biochar. This nitrogen doping enhances the surface reactivity and adsorption capacity of the biochar, especially toward certain organic compounds, such as CH_4_, CH_2_Cl_2_, C_2_H_4_, HCHO, and C_2_H_5_OH [39].

**Figure 3:**
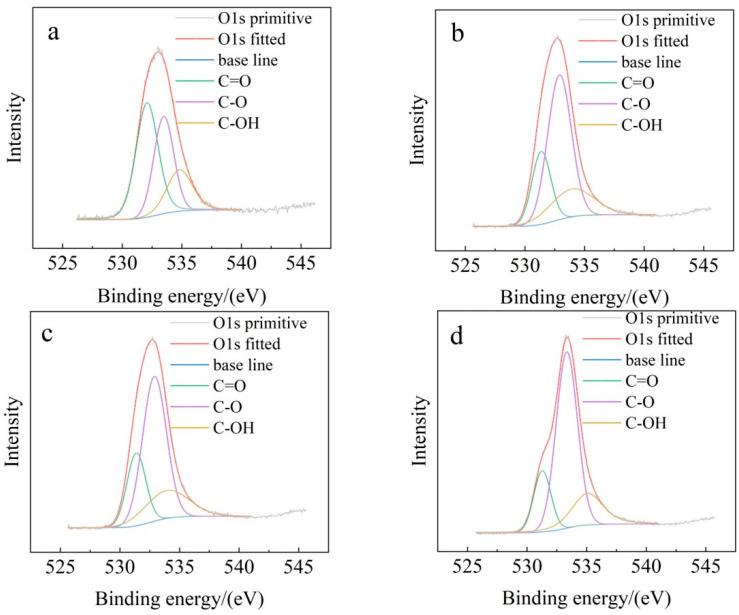
O1s spectra of SB: (**a**) Zn-Cl-SB; (**b**) Na-Cl-SB; (**c**) Na-S-SB; (**d**) Na-Cl/S-SB.

According to the results shown in Figure 3, SB mainly exhibits three types of bonds: the absorption peak at 531 eV corresponds to C=O bonds (representing O=C–O groups and amides), and at 533 eV, it corresponds to C–O bonds (representing ethers, esters, and alcohols) [40]. Compared to Zn-Cl-SB, the C=O content in all sodium-carbonate-activated SB samples is relatively lower. Conversely, the proportion of oxygen-containing functional groups, particularly C–O, increases. As shown by the FTIR data presented later, the results indicate that after sodium carbonate activation, the oxygen-containing functional groups on the biochar surface undergo changes, which is consistent with these findings. Generally, the C–O functional groups on the SB surface contribute significantly to improving its ability to adsorb dissolved organic matter in wastewater [34].

**Figure 4:**
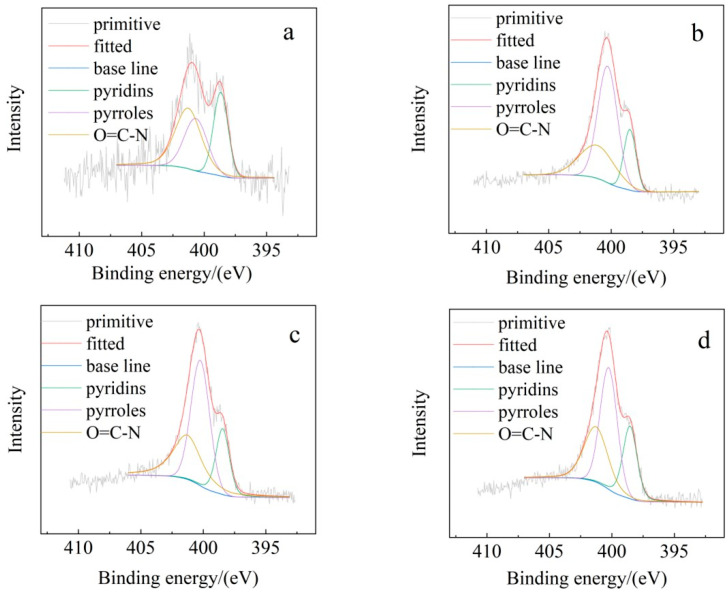
N1s spectra of SB: (**a**) Zn-Cl-SB; (**b**) Na-Cl-SB; (**c**) Na-S-SB; (**d**) Na-Cl/S-SB.

As shown in Figure 4, SB contains various forms of nitrogen-containing functional groups, including pyridine-like, pyrrole, pyridine, amide, imide groups, protonated pyridine and pyrrole, and amine salts. Figure 4 shows that SB mainly exhibits three types of nitrogen-containing functional groups: the absorption peak at 398.7 eV corresponds to pyridine functional groups, the peak at 400 eV corresponds to pyrrole functional groups, and the peak at 401 eV corresponds to amide structures [41]. Overall, the rearrangement of nitrogen-containing functional groups caused the N1s peak to shift towards higher binding energies and increased the proportion of pyrrole functional groups. Studies have shown that pyrrole functional groups exhibit more vigorous interaction intensity and better physical adsorption capacity for volatile organic compounds compared to pyridine functional groups [39].

#### 3.2.3. Functional Group

**Figure 5:**
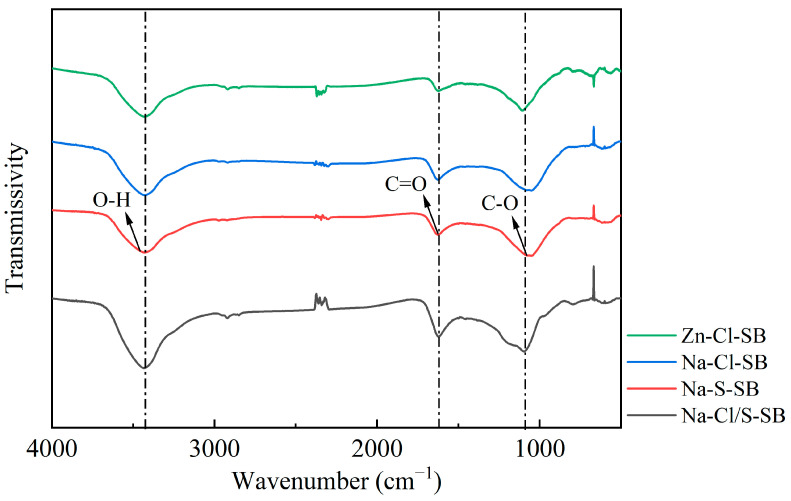
Fourier-transform infrared (FTIR) spectra of SB prepared by different methods.

As shown in Figure 5, SB samples prepared using different methods exhibit prominent stretching vibration absorption bands near 3425 cm^−1^ (range of 3200–3600 cm^−1^), mainly attributed to O–H stretching vibrations of hydroxyl, phenolic hydroxyl, and carboxyl functional groups on the biochar surface. Compared to the Zn-Cl-SB sample, Na-Cl-SB, Na-S-SB, and Na-Cl/S-SB exhibit a more pronounced absorption peak in the range of 1500–1710 cm^−1^, indicating a higher concentration or more developed structures of functional groups, such as C=C bonds, C=O groups, aromatic rings, and nitrogen-containing species (e.g., pyridine-like —C=N bonds and lactam-type N— bonds), resulting from sodium carbonate activation. Moreover, Na-Cl-SB, Na-S-SB and Na-Cl/S-SB show broader and more pronounced absorption peaks in the range of 1000–1300 cm⁻^1^, which consist of a series of C—O stretching vibration absorption bands, and these can be attributed to carboxylic acids, lactones, phenols, and ethers [42]. Furthermore, the spectrum of the Na-Cl/S-SB sample exhibits the most significant variation, indicating that Na-Cl/S-SB is rich in oxygen-containing functional groups because introducing the Na_2_CO_3_ activating agent allows for better penetration into the internal pores of the sludge and ensures sufficient contact with both the surface and internal structures. This process enables the organic matter within the sludge to be dispersed effectively. The alkaline chemical reactions of Na_2_CO_3_ contribute to the formation of diverse oxygen-containing functional groups, such as hydroxyl, carbonyl, and carboxyl contribute to chemical adsorption by providing active adsorption sites, strengthening the ability to capture contaminants of biochar [23].

#### 3.2.4. Crystal Structure

**Figure 6:**
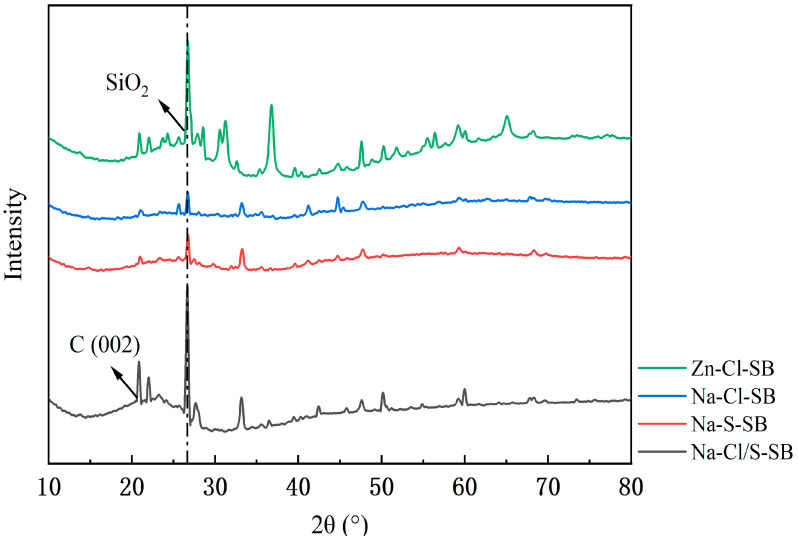
XRD spectra of SB prepared by different methods.

In this study, XRD was used to analyze the effect of different preparation methods on the crystalline structure of SB. As shown in Figure 6, Zn-Cl-SB exhibits prominent diffraction peaks at 2θ equals 27° and 37°, corresponding to the diffraction peaks of the SiO_2_ crystalline phase. In contrast, Na-Cl-SB and Na-S-SB show almost no impurity peaks. Na-Cl/S-SB also displays prominent diffraction peaks at 2θ equals 27° and 33°, corresponding to the diffraction peaks of the SiO_2_ crystalline phase, with fewer impurity peaks. Additionally, the diffraction peak of the C (002) plane in Na-Cl/S-SB indicates the presence of amorphous carbon, characterized by the absence of a refined peak structure. Compared to the highly ordered silica structures formed in Na-Cl-SB and Na-S-SB, the amorphous carbon structure in Na-Cl/S-SB, lacking a fixed crystal arrangement, provides more adsorption sites and exhibits a larger specific surface area and a more extensively developed pore structure [43]. This random structure and porosity enhance the interaction between the adsorbent and adsorbate, improving the adsorption capacity of the material. The formation of this structure is primarily attributed to the acid modification, which effectively etches the carbon material and promotes the creation of pores. The use of combined acids further enhances the pore-forming process, forming a large number of micropores and mesopores, thereby increasing the specific surface area [44]. Based on these microstructural characterization results, it can be concluded that Na-Cl/S-SB possesses a higher specific surface area, as well as abundant nitrogen-containing functional groups and oxygen-containing functional groups. Thus, Na-Cl/S-SB is believed to have excellent potential for adsorption performance.

### 3.3. Adsorption Performance for Wool Scouring Wastewater

#### 3.3.1. Organic Matter Adsorption Kinetic Analysis

**Figure 7:**
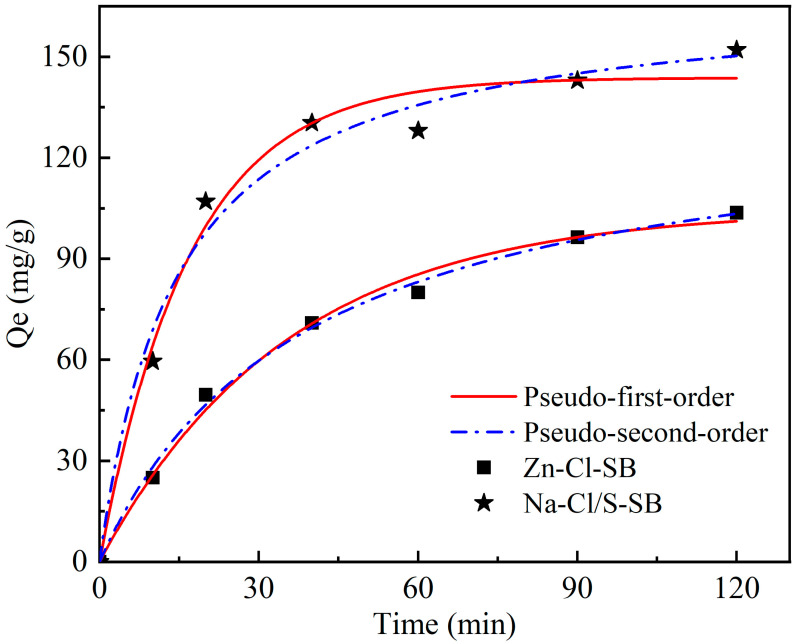
Kinetic fitting curves of Na-Cl/S-SB and Zn-Cl-SB for organic matter adsorption in wool scouring wastewater.

To explore the organic adsorption performance of the novel SB for wool scouring wastewater, the organic matter adsorption behavior of Na-Cl/S-SB and Zn-Cl-SB was analyzed using a kinetic model fitting. As shown in Figure 7 and Table 4, the adjusted correlation coefficients (R^2^_Adj_) for the PSO kinetic model of Na-Cl/S-SB and Zn-Cl-SB are 0.98978 and 0.9954, respectively, both higher than those for the PFO kinetic model. This indicates that the adsorption of organic matter in wool scouring wastewater by Na-Cl/S-SB and Zn-Cl-SB follows the PSO kinetic model. It can be inferred that the adsorption process of Na-Cl/S-SB for organic matter is primarily driven by chemical adsorption. According to the PSO kinetic model fitting results, the maximum adsorption capacity of Na-Cl/S-SB for organic matter in wool scouring wastewater was 168.3 mg/g, which was significantly higher than Zn-Cl-SB of 134.1 mg/g. Sun et al. also highlighted that activation methods significantly affect biochar’s adsorption capacity, which is consistent with our BET and SEM results showing that Na-Cl/S-SB possesses a more developed pore structure [33]. Table 2 presents the adsorption capacities of biochar activated with different agents, where Na-Cl/S-SB exhibits the highest adsorption capacity, demonstrating its superior ability for organic pollutant removal. These results show that Na-Cl/S-SB exhibits superior adsorption performance for organic matter in wool scouring wastewater.

#### 3.3.2. UV-Vis Spectra Analysis

**Figure 8:**
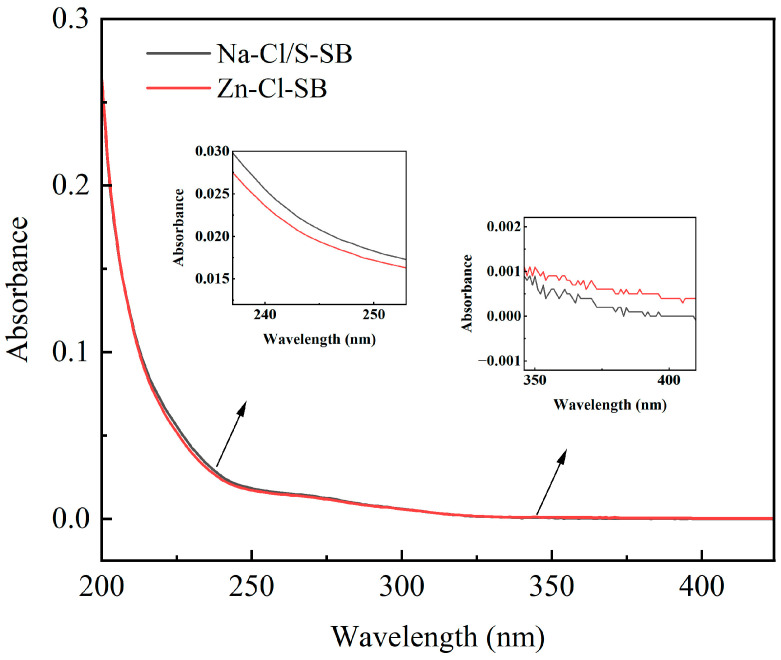
UV–vis absorption spectra of wool scouring wastewater after adsorption by Na-Cl/S-SB and Zn-Cl-SB.

To explore the adsorption performance of Na-Cl/S-SB and Zn-Cl-SB on DOM in wool scouring wastewater, UV-vis spectroscopy was used to evaluate the transformation of DOM in wastewater by analyzing absorbance variations at specific wavelengths. Additionally, UV spectral indices (E2/E3, E2/E4) were used to further examine the distribution characteristics of organic matter [45]. As shown in Figure 8, the wastewater treated with Na-Cl/S-SB and Zn-Cl-SB exhibited distinct absorption characteristics within the 200–400 nm wavelength range. In the short-wavelength region (240–250 nm), the absorbance of Na-Cl/S-SB-adsorption wastewater was slightly higher than that of Zn-Cl-SB-adsorption wastewater, indicating that Na-Cl/S-SB adsorption increased in low-molecular-weight dissolved organic matter. In contrast, in the long-wavelength region (350–400 nm), the absorbance of Na-Cl/S-SB-treated wastewater was significantly lower than that of Zn-Cl-SB, suggesting that Na-Cl/S-SB was more effective in removing high-molecular-weight humic substances [35]. As shown in Table 5, the E2/E3 index (A250/A365) negatively correlates with the molecular weight of organic matter. After Na-Cl/S-SB adsorption, E2/E3 increased from 1.77 to 6.10, indicating a significant decrease in the molecular weight of organic matter, with large organic molecules being removed and the proportion of small organic molecules increasing. The E2/E4 index (A240/A420) negatively correlates with the degree of humification. After Na-Cl/S-SB adsorption, E2/E4 increased from 233 to 255, indicating that the degree of humification decreased, meaning that the content of high molecular weight humic substances was reduced. Compared to Zn-Cl-SB, Na-Cl/S-SB exhibited a greater ability to remove high-molecular-weight and highly humified DOM while simultaneously increasing the proportion of small molecular DOM, which is more conducive to sub-sequent biodegradation. These findings suggest that Na-Cl/S-SB is a more effective adsorbent for DOM removal in wool scouring wastewater.

#### 3.3.3. EEM Spectra Analysis

**Figure 9:**
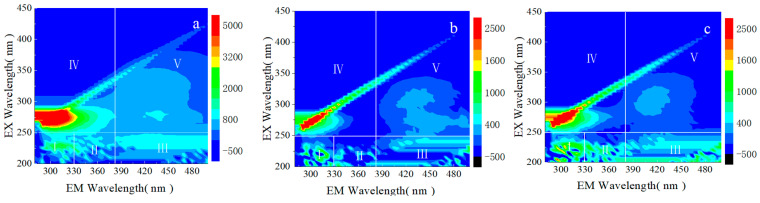
EEM analysis of dissolved organic matter in wool scouring wastewater: (**a**) raw wool scouring wastewater; (**b**) after Na-Cl/S-SB adsorption; (**c**) after Zn-Cl-SB adsorption.

EEM spectroscopy was used to analyze the impact of SB adsorption on the composition of DOM in the wastewater. As shown in Figure 9, the three-dimensional fluorescence spectra can be divided into five regions, corresponding to five categories of dissolved organic matter: Region I represents the first type of aromatic protein-like substances, Region II represents the second type of aromatic protein-like substances, Region III represents fulvic acid-like substances, Region IV represents protein with benzene rings and soluble microbial by-products, and Region V represents humic acid-like substances [46]. Figure 9a shows that the dissolved organic matter in wool scouring wastewater mainly comprises proteins with benzene rings, soluble microbial by-products, the first type of aromatic protein-like substances, and humic acid-like substances. As shown in Figure 9b, after adsorption treatment with Na-Cl/S-SB, the fluorescence intensity of dissolved organic matter in Regions I, II, and IV decreased significantly, indicating effective removal of aromatic protein-like substances, proteins with benzene rings, and soluble microbial by-products. This demonstrates that Na-Cl/S-SB exhibits excellent adsorption performance for these dissolved organic substances. As shown in Figure 9c, compared to Zn-Cl-SB, the fluorescence intensity of Regions I, II, III, and IV is significantly reduced with Na-Cl/S-SB. This indicates that Na-Cl/S-SB exhibits a significantly higher adsorption capacity for dissolved organic matter in wool scouring wastewater than Zn-Cl-SB.

## 4. Conclusions

This study successfully developed a novel Na_2_CO_3_-activated SB and evaluated its adsorption performance for organic pollutants in wool scouring wastewater. Compared with traditional ZnCl_2_-activation, Na_2_CO_3_-activated significantly improved the physicochemical properties of SB, increasing oxygen-containing and nitrogen-containing functional groups, which are crucial for adsorption interactions. Considering the potential structural collapse at high temperatures, 600 °C was selected as the optimal carbonization temperature, ensuring a stable porous structure. The Na_2_CO_3_ activation process increased the residual weight percentage of biochar carbonized material by 170%. Furthermore, the combined modification with HCl and H_2_SO_4_ significantly enhanced the specific surface area (509.3 m^2^/g), shifted the pore size distribution toward micropores and mesopores, improved the micropore volume, and optimized the crystalline structure of SB. Na-Cl/S-SB exhibited excellent adsorption performance for organic matter in wool scouring wastewater, with a maximum adsorption capacity of 168.3 mg/g. Furthermore, Na-Cl/S-SB demonstrated a superior ability to remove high-molecular-weight and highly humified DOM while simultaneously increasing the proportion of small molecular DOM, which is more conducive to subsequent biodegradation. The adsorption process followed the pseudo-second-order kinetic model, effectively removing macromolecular organic compounds such as aromatic protein-like substances, proteins with benzene rings, and soluble microbial by-products. Overall, this study explores the development of an innovative biochar through Na_2_CO_3_ activation and composite acid modification, aiming to enhance its structural properties and adsorption performance. Na_2_CO_3_ activation effectively enhances the specific surface area, optimizes the pore structure, and introduces beneficial functional groups while offering advantages in terms of environmental sustainability and scalability for large-scale production. Future research should focus on the regeneration and reuse of Na-Cl/S-SB, as well as its application in treating other complex wastewater types, to further expand its practical implications in environmental remediation.

## Figures and Tables

**Table 1:** Specific surface area, pore volume, pore size distribution and pore properties of SB.

Sample	BET Specific Surface Area/m^2^·g^−1^	Specific Surface Area of Micropores/m^2^·g^−1^	Total Pore Volume /cm^3^·g^−1^	Micropore Volume/cm^3^·g^−1^	Average Pore Size/nm	Average Mesopore Size/nm	Micropore Volume /%
Zn-Cl-SB	445.0	102.4	0.40	0.054	3.551	5.225	13.5
Na-Cl-SB	340.0	113.0	0.40	0.061	4.669	7.349	15.3
Na-S-SB	431.8	142.1	0.47	0.076	4.337	9.396	16.2
Na-Cl/S-SB	509.3	184.1	0.51	0.099	3.978	7.896	19.4

**Table 2:** Maximum organic adsorption capacities of various biochar.

Activator	Material	BET Specific Surface Area/m^2^·g^−1^	Organic Adsorption Capacity (mg/g)	Reference
H_2_SO_4_	Biochar	339.2	36.9	[2]
KMnO_4_	Biochar	238.7	29.7	[2]
Na_2_CO_3_ (Na-Cl/S-SB)	Sludge-based biochar	509.3	168.3	This study

**Table 3:** C1s, O1s, and N1s contents of SB.

Sample	O1s Content of Elements %	C1s Content of Elements %	N1s Content of Elements %	S2 and Cl2s Content of Elements %
Zn-Cl-SB	14.86	82.03	2.47	0.64
Na-Cl-SB	20.24	74.47	4.91	0.38
Na-S-SB	19.18	74.62	5.6	0.6
Na-Cl/S-SB	21.64	73.26	4.86	0.25

**Table 4:** Kinetic model parameters for the adsorption of organic matter in wool scouring wastewater by Na-Cl/S-SB and Zn-Cl-SB.

Kinetic Models	Parameters	Na-Cl/S-SB	Zn-Cl-SB
Pseudo-First-Order	*q*_1_ (mg g^−1^)	143.8 ± 4.59	104.7 ± 3.55
	*k*_1_ (min^−1^)	0.028	0.059
	R^2^	0.98434	0.99351
	R^2^_Adj_	0.98121	0.99221
Pseudo-Second-Order	*q*_2_ (mg g^−1^)	168.3 ± 8.21	134.1 ± 5.28
	*k*_2_ (g mg^−1^ min^−1^)	0.00041	0.00019
	R^2^	0.98411	0.99617
	R^2^_Adj_	0.98978	0.9954

**Table 5:** UV-vis spectral index of molecular weight of organic matter in wool scouring wastewater after Na-Cl/S-SB and Zn-Cl-SB adsorption.

Index of	Wastewater from Raw Wool Spinning	After Zn-Cl-SB Adsorption	After Na-Cl/S-SB Adsorption
E2/E3	1. 77	2.46	6.10
E2/E4	233	236	255

## Data Availability

Dataset available on reasonable request.

## Abbreviations

The following abbreviations are used in this manuscript:

SB	Sludge-based biochar
MLSSs	Mixed liquor suspended solids
MLVSSs	Mixed liquor volatile suspended solids
COD	Chemical oxygen demand
NDRC	National development and reform commission
PFASs	Perfluoroalkyl and polyfluoroalkyl substances
TGA	Thermogravimetric analysis
DTG	Derivative thermogravimetry
BET	Brunauer–Emmett–Teller
XRD	X-ray diffraction
XPS	X-ray photoelectron spectroscopy
FTIR	Fourier-transform infrared spectroscopy
EEM	Excitation–emission matrix spectroscopy
DOM	Dissolved organic matter
PFO	Pseudo-first-order
PSO	Pseudo-second-order
